# Secondary prevention of early-onset sepsis: a less invasive Italian approach for managing neonates at risk

**DOI:** 10.1186/s13052-018-0515-8

**Published:** 2018-06-28

**Authors:** Alberto Berardi, Chryssoula Tzialla, Laura Travan, Jenny Bua, Daniele Santori, Milena Azzalli, Caterina Spada, Laura Lucaccioni, on behalf of the GBS Prevention Working Group of Emilia-Romagna, L. Memo, G. Nicolini, M. Ciccia, A. Bastelli, F. Sandri, S. Ambretti, M. G. Capretti, L. Corvaglia, A. Dondi, M. Lanari, L. Pasini, L. Ragni, A. Albarelli, V. Fiorini, C. Giugno, P. Lanzoni, E. Di Grande, A. Polese, M. C. China, V. Rizzo, M. Stella, A. Zucchini, L. Malaguti, M. Azzalli, G. Garani, C. Lama, S. Nasi, P. Bacchini, G. Fragni, P. Baldassarri, R. M. Pulvirenti, E. Valletta, V. Venturoli, C. Alessandrini, M. L. Bidetti, S. Storchi Incerti, C. Di Carlo, A. Lanzoni, L. Serra, D. Silvestrini, A. Berardi F. Facchinetti, F. Ferrari, L. Lugli, C. Venturelli, M. Sarti, A. Volta, I. Dodi, L. Gambini, C. Magnani, B. Guidi, M. Bertelli, G. Biasucci, R. Chiarabini, N. De Paulis, D. Padrini, S. Riboni, M. F. Pedna, V. Sambri, L. Casadio, F. Marchetti, C. Muratori, G. Piccinini, C. Renzelli, S. Amarri, L. Baroni, E. Carretto, S. Fornaciari, G. Gargano, S. Pedori, M. Riva, C. Zuelli, G. Ancona, S. Bolognesi, I. Papa, G. Vergine, L. Viola, C. Chiossi, R. Pagano, C. Rivi, C. Zanacca, C. Bonvicini, R. Palmieri

**Affiliations:** 1 Unità Operativa di Terapia Intensiva Neonatale, Dipartimento Integrato Materno-Infantile, Azienda Ospedaliero-Universitaria Policlinico, Modena, Italy; 2 Neonatologia, Patologia Neonatale e Terapia Intensiva Neonatale, Fondazione IRCCS Policlinico “San Matteo”, Pavia, Italy; 3 Unità Operativa di Terapia Intensiva Neonatale, IRCCS “Burlo Garofolo”, Trieste, Italy; 4 Struttura Complessa di Pediatria e Neonatologia, Azienda Ospedaliera Santa Maria degli Angeli, Pordenone, Italy; 5 Unità Operativa di Terapia Intensiva Neonatale, Ospedale S. Anna, Ferrara, Italy; 6 Scuola di specializzazione in Pediatria, Università di Modena e Reggio Emilia, Azienda Ospedaliero-Universitaria Policlinico, Via del Pozzo, 71 -, 41124 Modena, MO Italy

**Keywords:** Group B streptococcus, Neonatal sepsis, Intrapartum antibiotic prophylaxis, Newborn infant, Risk factors, Prevention

## Abstract

Strategies to prevent early-onset sepsis (EOS) have led to a substantial decline in many countries. However, one of the most controversial topics in neonatology is the management of asymptomatic full-term and late preterm neonates at risk for EOS, and guidelines lack substantial consensus regarding this issue. A strategy for managing neonates, entirely based on serial physical examinations, has been developed in two Italian regions. This strategy seems safe, while reducing laboratory tests and unnecessary antibiotics. In the current commentary we provide area-based data concerning the prevention of EOS in 2 northern Italian regions, and we detail the results of their strategy for managing healthy-appearing newborns at risk for EOS.

## Background

Group B streptococcus (GBS) remains a leading cause of early-onset sepsis (EOS, the disease occurring at age 0–6 days) [1]. Intrapartum antibiotic prophylaxis (IAP) is the mainstay of prevention. At the time the first CDC guidelines were issued in the USA during the ‘90s, cases of GBS-EOS were 3–4/1000 live births, but currently they have declined to fewer than 0.25 per 1000 live birth [2]. However, guidelines for preventing EOS lack substantial consensus regarding the management of asymptomatic neonates at-risk for EOS. [2] CDC guidelines recommend full diagnostic evaluation and antibiotic therapy if signs of sepsis; limited evaluation and antibiotic therapy in case of chorioamnionitis; limited evaluation and observation if preterm birth of prolonged rupture of membrane [1]. However, ancillary laboratory tests have poor predictive value, and may result in medical intervention for a large number of uninfected infants [2]. A less invasive approach, entirely based on serial clinical observations is now recommended in some European guidelines [3].

## Main text

The recent national survey [4] shows that the screening-based strategy is largely prevalent and protocols in most centers are consistent with the CDC guidelines [1]. However, differences in GBS EOS sepsis occur among Northern, Central and Southern regions. Furthermore, 75% of respondent neonatal units would obtain laboratory testing and 91% would administer empirical antibiotics to full-term and preterm neonates exposed to inadequate IAP, although asymptomatic [4]. This practice is not consistent with the CDC guidelines [1]. Italian epidemiological data, on which to inform recommendations, are still insufficient. Furthermore there is no national laboratory-based surveillance system to tracking serotypes, incidence rates of GBS-EOS, and the impact of perinatal prevention interventions. Nevertheless, area-based data have been obtained prospectively in two northern Italian regions (Emilia-Romagna and Friuli-Venzia Giulia) [5–10]. In Emilia-Romagna, a detailed information has been obtained during a 9 years GBS surveillance period [6–10]. In both regions the prevention of EOS relies upon local protocols, mostly consistent with vagino-rectal screening and IAP according to the CDC guidelines (Table 1).

**Table 1:** Colonization rates and IAP in Emilia-Romagna and Friuli-Venezia Giulia

	Emilia-Romagna [6–10]		Friuli-Venezia Giulia [5]
Years in study	2003–2005	2009–2012	2005–2006
Live births	112,933	146,682	16,394
Preterm deliveries *(%)*	7.3	7.4	7.7
Prevention strategy	V-R screening	V-R screening	V-R screening
Rates of prenatal screening *(%)*	86.6	93.2 §	89.0 §
Rates of vagino-rectal cultures *(%)*	42.7	90.4 §	88.6
Rates of maternal GBS colonization *(%)*	18.1 §	21.4 §	19.8
Overall rates of IAP *(%)*	28.7	32.0	ND
Rates of IAP to women with GBS colonization *(%)*	92.6	90.2	83%
Prevalence of risk factors for EOS *(%)*	ND	20.1	22.1
GBS-EOS (cases/1000 live births)	0.25	0.15	0
GBS-EOS mortality (cases/1000 live births)	0.02	0.01	0
GBS-EOS mortality in full-term neonates, (cases/1000 live births)	0	0	0

*EOS* early-onset sepsis, *GBS* group B streptococcus, *IAP* intrapartum antibiotic prophylaxis, *V-R* vaginorectal

§ refers to full term deliveries

Colonization ranges from 18 to 21% of pregnant women, [5–10] and up to ~1/3 of them receive IAP. Area-based data on GBS-EOS is available in Emilia-Romagna (from 2003 onwards), and in Friuli-Venezia Giulia (from 2005 to 2006). In both regions incidence rates of GBS-EOS are currently under 0.25/1000 live births.

The case fatality ratio among full-term neonates is close to zero. Both regions recommend no laboratory tests or empirical antibiotics for neonates at-risk for EOS who have an entirely normal physical exam [5, 6]. Their strategy is based on serial physical examinations (SPE), i.e. on vital signs that can be easily detected by medical and non-medical staff. Each examiner fills in and signs at standard intervals a sheet (included in the medical records) detailing general wellbeing, reactivity, spontaneous motility, skin colour (including perfusion) and respiratory signs. Seven (at age 3–6–12–18-24–36–48 h) or 12 visits (at 1, 2, 4, 8, 12, 16, 20, 24, 30, 36, 42, and 48 h) are recommended in Emilia-Romagna and Friuli-Venezia-Giulia respectively. All neonates are left with their own mothers (rooming in), without being admitted to NICU or level II nursery. Every evaluation requires a maximum of 1 to 2 min; nursing staff and midwives give notification to clinicians when signs of illness develop.

During 2005–2006 Cantoni and co-workers compared in Friuli Venezia Giulia 7628 full term neonates managed with the standard approach to 7611 full term newborns managed through the SPE strategy. [5] Sepsis screenings and antibiotic treatments decreased significantly (from 6.3 to 0.5%, *p* < 0.01 and from 1.2 to 0.5%, *p* < 0.01 respectively). No treatment was delayed. Furthermore, a study carried out in a single centre of Emilia-Romagna reported significant reduction of laboratory tests (from 11.6 to 1.6%, *p* < 0.01) and empirical antibiotics (from 2.8 to 0.6%, *p* < 0.01) [11]. In a further retrospective study carried out in 3 NICUs of Emilia-Romagna, 2092 full-term and late preterm neonates were managed with SPEs. Among 632 initially asymptomatic neonates at-risk for EOS, only 3% were evaluated (0.9% of the entire cohort) and only 1.1% (0.3% of the entire cohort) were given empirical antibiotics. No cases of EOS were missed, and no neonates had complications or a worse outcome due to this strategy [12]. Although evidences supporting the SPE based prevention in regions with higher rates of GBS EOS are still lacking, we are convinced that it could be similarly effective.

## Discussion

Here we resume and compare area-based data from 2 Italian regions. They add information for assisting healthcare providers in planning national strategies. As preterm neonates younger than 34 weeks’ gestation need special care in NICUs, we focused on late preterm (34–36 weeks’ gestation) and full-term (≥37 weeks’ gestation) neonates, that account for over 90% of total deliveries and are managed in all birthing centers across country. The recent national survey [4] has shown that great resources are poored into prevention of EOS, and often are given unnecessary antibiotics. Ancillary laboratory tests currently available are low predictive for asymptomatic neonates and have insufficient accuracy for guiding the decision as to whether neonates should be treated with antibiotics [2].

A recent survey carried out in in Europe, North America, and Australia have shown that 14% of full-term neonates worldwide are evaluated annually for EOS. Furthermore, 8% of them are treated with antibiotics, although EOS is confirmed only in 0.1% [13]. The large number of uninfected newborns being evaluated and treated with antibiotics may lead to maternal/infant separation and longer length of stay. Furthermore, antibiotics can increase the risk of resistant pathogens and alter the neonatal microbiome, with risks of health problems in later life (allergies, diabetes, and inflammatory bowel diseases) [13, 14]. However, many infants with mild illness can be observed safely without treatment (unless clinical signs worsen or fail to improve), as they become asymptomatic over the first 6 h. Finally, in a recent and very comprehensive review on risks and benefits of evolving rule-out sepsis practices, [15] Hooven and co-workers estimate (number needed to harm, NNH) the potential complications of strategies for managing neonates at risk for EOS. They compare the rare risks of developing severe symptoms of EOS (NNH = 1610) and the more common risks of infiltrate after i.v. infusion (NNH = 7) or delayed breastfeeding (NNH = 2.9). Investigators conclude that there is strong evidence to support observation-based approaches for managing neonates at risk for EOS.

## Conclusion

The SPEs strategy has proven sensitive for timely detection of all cases of EOS, not only for GBS sepsis. By providing strong assurance that frequent examinations actually are performed, this strategy seems safe, reliable and easy to perform. Strategies reducing neonatal hospitalization during the first week life, respecting the bonding of mother and infant, and minimising unnecessary antibiotics must be taken into consideration.

## Abbreviations

EOS: Early-onset sepsis
GBS: Group B streptococcus
IAP: Intrapartum antibiotic prophylaxis
NICU: Neonatal intensive care unit
NNH: Number needed to harm
SPE: Serial physical examinations
